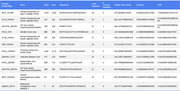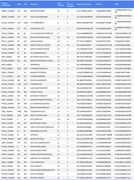# Identification of Disease‐Associated Epitopes in Alzheimer's Disease Using Unbiased Antibody Profiling

**DOI:** 10.1002/alz70855_106171

**Published:** 2025-12-24

**Authors:** Nathaniel J Barton, Qi Wang, Rebecca L. Best, Michael Jhatro, Kathy Kamath, John Shon, Diego Mastroeni, Elaine T Lim, Benjamin Readhead, Yingleong Chan

**Affiliations:** ^1^ UMass Chan Medical School, Worcester, MA, USA; ^2^ Arizona State University, Tempe, AZ, USA; ^3^ ASU‐Banner Neurodegenerative Disease Research Center, Tempe, AZ, USA; ^4^ ASU, Phoneix, AZ, USA; ^5^ Banner Sun Health Research Institute, Sun City, AZ, USA; ^6^ Arizona Alzheimer's Consortium, Tempe, AZ, USA; ^7^ Arizona Alzheimer's Consortium, Phoenix, AZ, USA

## Abstract

**Background:**

Alzheimer's disease (AD) is characterized by complex immune interactions, yet the role of antibody‐mediated responses remains poorly understood. Recent advances in high‐throughput epitope screening enable the identification of disease‐associated epitopes that may play a role in AD pathogenesis. We previously developed a computational pipeline to detect disease‐related epitopes from high‐throughput epitope profiling data, and extend this approach to AD to identify potential antibody responses to pathogenic or autoantigenic epitopes in cerebrospinal fluid (CSF) samples.

**Method:**

We performed IgG epitope profiling on CSF samples from 625 individuals, including 359 AD patients, 97 mild cognitive impairment (MCI) controls, and 158 cognitively normal controls. The remaining samples did not fit these categories and were excluded from subgroup analyses. CSF samples were screened using Serimmune's Serum Epitope Repertoire Analysis (SERA) platform, which profiles IgG binding to a random 12‐mer bacterial display library. Sequencing of enriched 12‐mers produced approximately 3 million reads per sample. K‐mers (k=5,6) were extracted, and enrichment scores were calculated based on expected amino acid distributions. Identified k‐mers were mapped to human and viral proteomes using protein tiling and permutation analysis to assess statistical enrichment. Epitopes were identified via peak calling, clustered using complete‐linkage clustering, and scored for disease association using an outlier sum statistic. Multiple hypothesis correction was performed using the Benjamini‐Hochberg method.

**Result:**

Comparison of AD patients to cognitively normal controls revealed 12 distinct epitopes from 12 viral proteins, spanning multiple viral families (Table 1). These epitopes originated from 9 viruses, with a notable overrepresentation of herpesviruses (HHV8P, HHV6U, EBVB9, HCMVM). Additionally, we identified 42 CNS‐expressed autoantigens associated with AD (Table 2). These included ion channels (SCN1A_HUMAN, SCN3A_HUMAN, ASIC4_HUMAN), neurotransmitter receptors (NTRK2_HUMAN, GRIA1_HUMAN), synaptic and neurodevelopmental proteins (SV2B_HUMAN, ASTN1_HUMAN), and immune‐regulatory proteins (PD1L1_HUMAN, P2Y10_HUMAN), suggesting a potential autoimmune component in AD pathology.

**Conclusion:**

Our computational approach successfully identifies putative AD‐associated epitopes, with a significant overrepresentation of herpesvirus‐derived epitopes and CNS autoantigens in AD cases. These findings support a growing body of evidence implicating viral infections and potential autoimmune mechanisms in AD pathology. Further validation and mechanistic studies are warranted to explore the potential role of these epitopes in AD progression.